# Production of recombinant 503 antigen of *Leishmania infantum chagasi *using cultivation in batch and fed-batch

**DOI:** 10.1186/1753-6561-8-S4-P186

**Published:** 2014-10-01

**Authors:** Michelle Rossana Ferreira Vaz, Francisco Canindé de Sousa Junior, Carlos Eduardo de Araújo Padilha, Daniella Regina Arantes Martins, Everaldo Silvino Santos, Gorete Ribeiro Macedo

**Affiliations:** 1 Centro de Desenvolvimento Sustentável do Semiárido, Universidade Federal de Campina Grande, Sumé, PB, 58540-000, Brazil; 2 Departamento de Engenharia Química, Universidade Federal do Rio Grande do Norte, Natal, RN, 59078-970, Brazil; 3 Departamento de Biologia Celular e Genética, Universidade Federal do Rio Grande do Norte, Natal, RN, 59078-970, Brazil

## Background

Visceral leishmaniasis is an infecto-parasitarian disease caused by obligatory intracellular protozoa belonging to the genus *Leishmania*, and might be lethal since there is no precocious diagnosis and proper treatment. Despite the considerable effort, there is no effective and safe vaccine for human use [1]. Genetically modified micro-organisms are used industrially in general for production of hormone, antibiotics and proteins. In the production of heterologous molecules, both the expression and the production step are important [2]. Therefore, the study of cultivation conditions in bioreactor plays an important role for the process viability of batch and fed-batch [3]. Thus, the aim of this work was to study strategies for production of the 503 antigen of *Leishmania infantum chagasi *using cultivation in batch and fed-batch.

## Methods

The strain of *E. coli *expressing 503 antigen of *Leishmania infantum chagasi *was kindly provided by Dr. Mary Wilson (University of Iowa, USA) [1]. The clone was cultured in 2xTY medium supplemented with ampicillin and kanamycin. The cultivations were carried out using a bench bioreactor with a work volume of 1.5 L, at frequency of agitation of 400 rpm and constant output aeration of 1 vvm. The expression of the recombinant protein was induced by the addition of lactose to the cultivation medium at the final concentration of 10 g/L [4]. Optimization of cultivation conditions of 503 antigen was performed in batch and fed-batch. Then, assays of the fractions were performed by Lowry method and electrophoresis, Biomass concentration was monitored by the dry weigh and the acetic acid concentration was assayed by high-performance liquid chromatography.

## Results and conclusions

Fed-batch culture is one of the most performed strategies to reach high cell densities of *E.coli *and consequently high recombinant protein productivities. It was observed that to the cultivations in fed-batch, the agitation of 400 rpm resulted increased the biomass of 2.5 g/L to 11 g/L. It was observed the same behavior for the protein using both batch and fed-batch (0.11 g/L). In the present study in the both processes it was not observed the inhibitory effect in the cellular growth as well as on the 503 protein expression. The highest acetic acid concentration was obtained at the agitation speed of 400 rpm (0.2 g/L) that occurred during the first two hours of cultivation. However, this concentration was inferior to 0.9 g/L that according to [5] have no inhibitory effect in the growth.
